# Elevated plasma levels of epithelial and endothelial cell markers in COVID-19 survivors with reduced lung diffusing capacity six months after hospital discharge

**DOI:** 10.1186/s12931-022-01955-5

**Published:** 2022-02-21

**Authors:** Oriol Sibila, Lídia Perea, Núria Albacar, Jorge Moisés, Tamara Cruz, Núria Mendoza, Belen Solarat, Gemma Lledó, Gerard Espinosa, Joan Albert Barberà, Joan Ramon Badia, Alvar Agustí, Jacobo Sellarés, Rosa Faner

**Affiliations:** 1 Pulmonary Service, Respiratory Institute, Hospital Clínic, University of Barcelona, C/Villaroel 170, 08036 Barcelona, Spain; 2 Institut d’Investigacions Biomèdiques August Pi i Sunyer (IDIBAPS), C/Roselló 149, 08036 Barcelona, Spain; 3 Centro de Investigación Biomédica en Red de Enfermedades Respiratorias (CIBER), Barcelona, Spain; 4 Autoimmune Diseases Department, IDIBAPS, University of Barcelona, Hospital Clínic, Barcelona, Spain

**Keywords:** Post-COVID, *Sequelae*, DLCO, Epithelial markers, Endothelial markers

## Abstract

**Background:**

Some COVID-19 survivors present lung function abnormalities during follow-up, particularly reduced carbon monoxide lung diffusing capacity (DLCO). To investigate risk factors and underlying pathophysiology, we compared the clinical characteristics and levels of circulating pulmonary epithelial and endothelial markers in COVID-19 survivors with normal or reduced DLCO 6 months after discharge.

**Methods:**

Prospective, observational study. Clinical characteristics during hospitalization, and spirometry, DLCO and plasma levels of epithelial (surfactant protein (SP) A (SP-A), SP-D, Club cell secretory protein-16 (CC16) and secretory leukocyte protease inhibitor (SLPI)), and endothelial (soluble intercellular adhesion molecule 1 (sICAM-1), soluble E-selectin and Angiopoietin-2) 6 months after hospital discharge were determined in 215 COVID-19 survivors.

**Results:**

DLCO was < 80% ref. in 125 (58%) of patients, who were older, more frequently smokers, had hypertension, suffered more severe COVID-19 during hospitalization and refer persistent dyspnoea 6 months after discharge. Multivariate regression analysis showed that age ≥ 60 years and severity score of the acute episode ≥ 6 were independent risk factors of reduced DLCO 6 months after discharge*.* Levels of epithelial (SP-A, SP-D and SLPI) and endothelial (sICAM-1 and angiopoietin-2) markers were higher in patients with reduced DLCO, particularly in those with DLCO ≤ 50% ref. Circulating SP-A levels were associated with the occurrence of acute respiratory distress syndrome (ARDS), organizing pneumonia and pulmonary embolisms during hospitalization.

**Conclusions:**

Reduced DLCO is common in COVID-19 survivors 6 months after hospital discharge, especially in those older than 60 years with very severe acute disease. In these individuals, elevated levels of epithelial and endothelial markers suggest persistent lung damage.

**Supplementary Information:**

The online version contains supplementary material available at 10.1186/s12931-022-01955-5.

## Background

About 20% of patients infected by SARS-CoV-2 require hospitalization for pneumonia (COVID-19) [1]. The epidemiological, pathophysiology and clinical characteristics of these patients during the acute phase of the disease have been extensively described [2], but potential long-term pulmonary *sequelae*, risk factors and underlying mechanisms in survivors remain still unclear. Previous studies showed that COVID-19 survivors may present lung function abnormalities both at hospital discharge [3] and at 3–6 months follow-up [4–10], particularly reduced carbon monoxide lung diffusion capacity (DLCO) which is observed in 20 to 80% of them. The clinical severity of COVID-19 [4, 5], radiologic extension [5], development of acute respiratory distress syndrome (ARDS) [4, 5] and pulmonary embolism [4, 8] during the acute episode increases the risk of abnormal DLCO during follow-up, but whether this DLCO impairment is temporary [9] or persistent over time [10] is unclear. Likewise, the mechanisms underlying reduced DLCO after hospital discharge are also unknown albeit they may likely include epithelial and/or endothelial dysfunction [11].

Several circulating biomarkers can reflect pulmonary epithelial and endothelial damage [12]. Surfactant proteins (SP), such as SP-A and SP-D, are pulmonary secreted proteins mainly by the alveolar type II epithelial cells (AEC-II) that leak into the bloodstream when the alveolocapillary barrier is damaged [13]. Other epithelial markers such as Club cell secretory protein-16 (CC16), a secretoglobin mainly produced by Club epithelial cells, and secretory leukocyte protease inhibitor (SLPI), one of the major protease inhibitors at mucosal surfaces, are found at high concentrations in plasma when pulmonary injury occurs [14, 15]. On the other hand, other circulating biomarkers, like soluble sE-selectin, Angiopoietin-2 and intercellular adhesion molecule 1 (sICAM-1), reflect activated or damaged endothelium [16]. In fact, previous reports showed that during the acute phase of COVID-19, sE-selectin, Angiopoietin-2 and sICAM-1 are associated with disease severity [17–19]. In survivors of COVID-19, these epithelial markers have not been investigated so far and their potential role in relation to long-term pulmonary *sequelae* is unknown.

We hypothesize that severe COVID-19 damage epithelial and/or endothelial lung cells and that such damage persists in patients with reduced DLCO after hospital discharge. To test this hypothesis, we compared the clinical characteristics, risk factors and circulating levels of epithelial and endothelial markers 6 months after hospital discharge in survivors of COVID-19 with normal or reduced DLCO.

## Methods

### Study design, participants and ethics

**Fig. 1:**
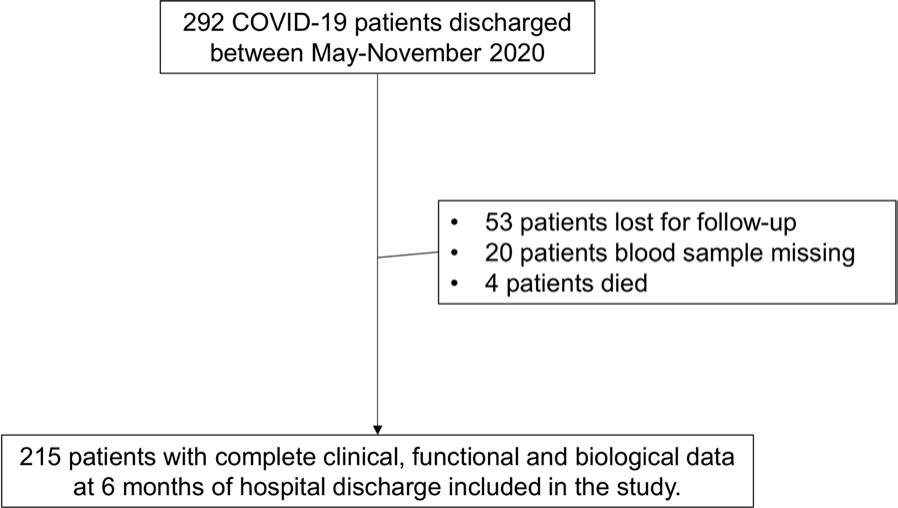
Study flow-chart

This prospective, observational study included 215 adults who were hospitalized in our institution between May and November 2020 because of PCR-confirmed COVID-19 pneumonia and studied at 6 months after hospital discharge. Figure 1 presents the consort diagram of the study. STROBE guidelines were used to ensure the reporting of this observational study [20]. The study protocol was approved by the Ethical Review Board of our hospital (HCB/2020/0422), and all patients signed their informed consent.

### Measurements

Demographic, clinical and biological characteristics were recorded on hospital admission and at 6 months after discharge. During admission, patients were treated according to international recommendations [21, 22]. The severity of the acute disease was determined according to the seven-category severity scale recommended by WHO [21]: score 3: admitted to the hospital but not requiring supplemental oxygen; score 4: requiring oxygen by mask or nasal prongs; score 5: requiring non-invasive ventilation or high-flow oxygen; score 6: requiring intubation or mechanical ventilation; and score 7: requiring ventilation plus additional organ support—pressors, renal replacement therapy (RRT), extracorporeal membrane oxygenation (ECMO). ARDS was defined by the Berlin criteria [23]. Organizing pneumonia was defined as the presence of different degrees of bilateral ground glass opacities with peripheral, linear and perilobular consolidations, some of them with reverse halo appearance, associated with bronchial dilatation and architectural distortion [24].

All patients followed the current Spanish Society of Pulmonology and Thoracic Surgery (SEPAR) consensus for post-COVID-19 clinical follow-up [25]. In all patients, spirometry and DLCO were measured (Medisoft, Sorinnes, Belgium) 6 months after discharge following international recommendations [26, 27] adapted to the current pandemic situation [28, 29]. Reference values were those of a Mediterranean population [30].

Blood was collected in EDTA tubes by peripheral venepuncture 6 months after discharge and was then centrifuged at 600xg 10 min 4ºC to obtain the plasma which was stored at -80ºC until analysis. Plasma CC16, SP-D (Cloud-Clone Corp, TX, USA), SP-A (Novus Biologicals, CO, USA), SLPI, Angiopoietin-2, sICAM-1 (R&D Systems, Minneapolis, MN, USA) and sE-selectin (RayBiotech, Peachtree Corners, GA) levels were measured by validated commercially available ELISA kits following manufacturer instructions. Plasma samples were diluted 1/10 for CC16, SP-D, SP-A and Angiopoietin-2, 1/80 for SLPI, 1/25 for sE-selectin and 1/20 for sICAM-1. The limits of detection were 0.156 ng/ml for CC16, 0.625 ng/ml for SP-D, 15.625 pg/ml for SP-A, 62.5 pg/ml for SLPI, 24.7 pg/ml for sE-selectin, 1.6 ng/ml for sICAM-1 and 46.9 pg/ml for Angiopoietin-2.

### Statistical analysis

Participants were categorized according their DLCO values at 6 months after discharge as normal (≥ 80% ref.) or low (< 80% ref.); the latter were further subdivided as moderate (80–50% ref.) or severe (< 50% ref.). Results are presented as n, frequency, mean ± standard deviation (SD), median [interquartile range -IQR]. Groups were compared using ANOVA, Student t-test, χ^2^. Fisher exact test, Kruskal–Wallis or Mann–Whitney tests, as appropriate according to the normal data distribution Multiple comparisons between groups were analyzed by the Dunn’s test. Bivariate correlations were analysed using the Spearman rank test. Multivariable adjusted logistic regression models were used to investigate risk factors of low DLCO (< 80% ref.), with age, hypertension, smoking status (never, current, former) and WHO acute disease severity score as independent variables. A p value < 0.05 was considered significant. Analyses were performed using R version 3.6.1, SPSS version 20 (IBM Inc, Armonk, NY) or GraphPad Prism 7 (GraphPad Software, Inc, La Jolla, Calif).

## Results

### Cohort characteristics

Mean age was 61.4 ± 11.8 years, and 66% of patients were male. The most frequent comorbid condition prior to hospitalization was arterial hypertension (40%). Most frequent symptoms on admission were fever (73%), cough (59%) and dyspnoea (46%). 44% of patients were admitted to the ICU, and 22% required mechanical ventilation. ARDS was observed in 35% of patients, while organizing pneumonia and pulmonary embolism occurred in 50% and 7% respectively during hospital admission. Length of hospital stay was 21.3 ± 18.6 days.

**Fig. 2:**
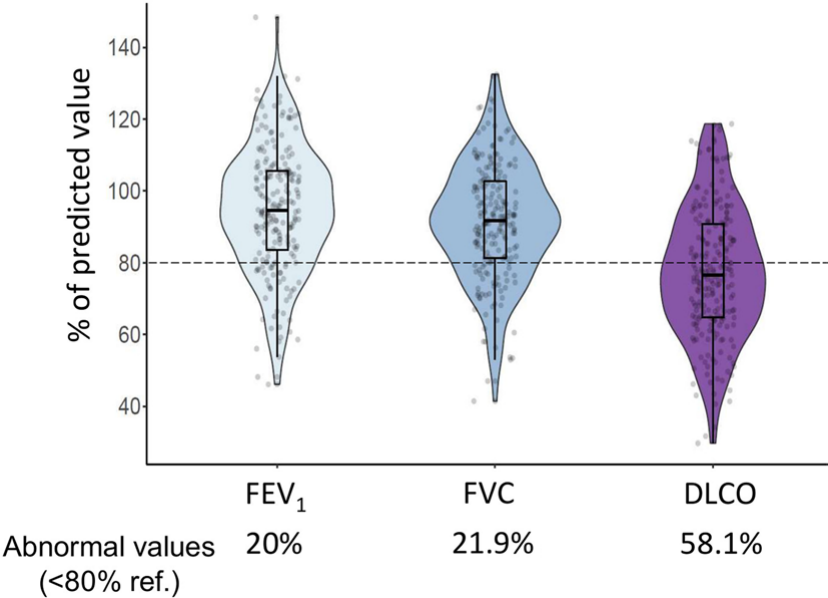
Proportion of patients with abnormal pulmonary values (< 80% ref.). Values of FEV_1_, FVC or DLCO at 6-months after hospital discharge. For further explanations, see text

At 6 months follow-up, 52% of patients remained symptomatic, predominantly with dyspnoea (26%) and fatigue (18%). On average, spirometry was normal (Forced expiratory volume in 1^st^ second (FEV_1_) 94.7 [83.5–105.8] % ref, Forced Vital Capacity (FVC) 91.9 [81–103] % ref., and FEV_1_/FVC 79.1 [73.9–83]). Mean DLCO was 76.6 [64.7–91] % ref. Collectively, 136 (63.7%) patients had one or more abnormal lung function value. Specifically, 43 patients (20%) had abnormal FEV_1_, 47 patients (21.9%) had abnormal FVC and 125 patients (58.1%) had abnormal DLCO values (% ref) (Fig. 2). Among patients with abnormal DLCO, 90 (42%) had a moderate reduction (50–80% ref.) and 35 (16%) a severe one (≤ 50% ref.).

### Comparison of patients with normal vs*.* reduced DLCO 6 months after hospital discharge

Table 1 shows that patients with reduced DLCO 6 months after hospital discharge were older, more frequently smokers and suffered more arterial hypertension before admission for the acute COVID-19 episode. During hospital admission they had higher levels of C-reactive protein, lactate dehydrogenase (LDH) and circulating leukocytes, suffered more severe disease and required mechanical ventilation more often. Their length of ICU and hospital stay was longer. Six months after hospital discharge, they showed persistent dyspnoea and cough more often, and their mean levels of LDH and circulating leukocytes remained higher. Interestingly, 35 patients (58%) from score 3 and 4 severity disease had reduced DLCO 6 months after hospital discharge without suffering from respiratory diseases nor smoking.

**Table 1:** Comparison of COVID-19 patients with normal or abnormal DLCO values 6 months after hospital discharge

	DLCO ≥ 80% ref N = 90 (41.9%)	DLCO < 80% ref N = 125 (58.1%)	*p-*value
*Demographics and clinical characteristics*			
Male (n, %)	59 (65.6)	71 (56.8)	0.2
Age (years)	**56.9 ± 12.7**	**65.3 ± 10.4**	** < 0.0001**
Smoking (n, %)			**0.02**
Never	**71 (78.9)**	**77 (61.6)**	
Current	**1 (1.1)**	**6 (4.8)**	
Former	**18 (20)**	**42 (33.6)**	
Comorbidities, (n, %)			
Hypertension	**26 (28.9)**	**59 (47.2)**	**0.007**
Diabetes	5 (5.6)	15 (12)	0.2
Cardiovascular disease	8 (8.9)	22 (17.6)	0.07
Asthma	5 (5.6)	8 (6.4)	1
COPD	2 (2.2)	7 (5.6)	0.3
Hepatic disease	5 (5.6)	5 (4)	0.7
Solid neoplasm	3 (3.3)	5 (4)	1.0
*Acute COVID-19 (in hospital)*			
Symptoms, (n, %)			
Any one of the following symptoms	75 (83.3)	98 (78.4)	0.4
Fever	70 (77.8)	87 (69.6)	0.2
Cough	57 (63.3)	70 (56)	0.3
Dyspnoea	38 (42.2)	60 (48)	0.4
Joint Pain	23 (25.6)	24 (19.2)	0.3
Diarrhoea	21 (23.3)	20 (16)	0.2
Sputum production	10 (11.1)	16 (12.8)	0.7
Headache	**13 (14.4)**	**5 (4)**	**0.01**
Chest Pain	11 (12.2)	7 (5.6)	0.1
Biomarkers			
CRP (mg/dL)	**6.7 [3.6–14.5]**	**10.4 [6.4–20]**	**0.003**
D-dimer (ng/mL)	1015 ± 1470	1493 ± 2152	0.1
Ferritin (ng/mL)	892.7 ± 862.1	1026.8 ± 987.7	0.5
LDH (U/L)	**303 [240–378]**	**362 [287–500]**	**0.002**
Creatinine (mg/dL)	0.8 ± 0.2	1 ± 0.6	0.06
Platelets (10^9^/L)	205.9 ± 87.5	242.8 ± 270.4	0.3
Leukocytes (10^9^/L)	**5.8 ± 2.3**	**6.9 ± 3.9**	**0.04**
Lymphocytes (10^9^/L)	0.9 ± 0.4	0.8 ± 0.5	0.6
Severity of disease (WHO), (n, %)			
Score 3	**26 (28.9)**	**23 (18.4)**	**0.04**
Score 4	**29 (32.2)**	**37 (29.6)**	
Score 5	**24 (26.7)**	**29 (23.2)**	
Score 6	**6 (6.7)**	**21 (16.8)**	
Score 7	**5 (5.6)**	**15 (12)**	
Hospitalization (n, %)			
ICU admission	34 (37.8)	61 (48.8)	0.1
MV	**11 (12.2)**	**36 (28.8)**	**0.004**
NIMV	6 (6.7)	15 (12)	0.2
ARDS	**22 (24.4)**	**53 (42.4)**	**0.006**
Organizing pneumonia	39 (43.3)	68 (54.4)	0.1
Pulmonary embolism	3 (3.3)	12 (9.6)	0.1
Length of hospital stay	**13**	**19 **	** < 0.0001**
Length of ICU stay	**7 **	**18 **	**0.002**
6 months after hospital discharge			
Symptoms, (n, %)			
Any one of the following symptoms	42 (46.7)	69 (55.2)	0.2
Dyspnoea	**14 (15.6)**	**42 (33.6)**	**0.003**
Fatigue	19 (21.1)	31 (24.8)	0.6
Cough	**7 (7.8)**	**23 (18.4)**	**0.03**
Joint Pain	6 (6.7)	15 (12)	0.2
Diarrhoea	1 (1.1)	3 (2.4)	0.6
Sputum production	5 (5.6)	12 (9.6)	0.3
Headache	8 (8.9)	8 (6.4)	0.6
Chest pain	10 (1.1)	9 (7.2)	0.3
Biomarkers			
CRP (mg/dL)	0.5 ± 0.2	0.5 ± 0.5	0.4
D-dimer (ng/mL)	360 ± 291	510 ± 998	0.4
Ferritin (ng/mL)	127 ± 102	113 ± 108	0.7
LDH (U/L)	**172 [155–190]**	**192 [172–209]**	**0.001**
Creatinine (mg/dL)	0.9 ± 0.2	1.1 ± 1.1	0.2
Platelets (10^9^/L)	227.9 ± 63	233.9 ± 74.5	0.7
Leukocytes (10^9^/L)	**6.2 ± 1.3**	**7.2 ± 2.6**	**0.02**
Lymphocytes (10^9^/L)	1.9 ± 0.6	2.2 ± 0.7	0.08

Data is presented as mean ± standard deviation or median [interquartile range]. Significantly different variables (p < 0.05) are highlighted using bold text

*COPD* chronic obstructive pulmonary disease, *ICU* intensive care unit, *LDH* lactate dehydrogenase, *CRP* protein C reactive, *MV* mechanical ventilation, *NIV* non-invasive mechanical ventilation

Table 2 presents the results of univariate and multivariate analyses of risk of abnormal DLCO at 6 months follow-up. Multivariate analysis showed that age ≥ 60 years (OR 2.92 [95CI 1.64–5.21], p < 0.001) and WHO severity score 6 (OR 3.03 [95CI 1.14–8.10], p = 0.02) were independent predictors of reduced DLCO after hospital discharge.

**Table 2:** Univariate and multivariate analysis for risk of abnormal DLCO at 6-months

	Univariate analysis			Multivariate analysis		
Variables	OR	CI 95%	p-value	OR	CI 95%	p-value
Age ≥ 60 years	**2.38**	**1.30–4.35**	**0.005**	**2.92**	**1.64–5.21**	** < 0.001**
Hypertension	1.63	0.87–3.06	0.13			
Former and current smokers	1.88	0.97–3.66	0.06			
Disease severity score 4	1.43	0.66–3.14	0.37			
Disease severity score 5	1.48	0.65–3.37	0.36			
Disease severity score 6	**4.00**	**1.31–12.16**	**0.015**	**3.03**	**1.14–8.10**	**0.027**
Disease severity score 7	3.70	1.10–12.46	0.035	2.95	0.99–8.77	0.052

Variables that remained significant (p < 0.05) in the multivariate analysis are highlighted in bold text

*OR* odds ratio, *CI 95%* 95% confidence interval

### Epithelial and endothelial biomarkers

**Fig. 3:**
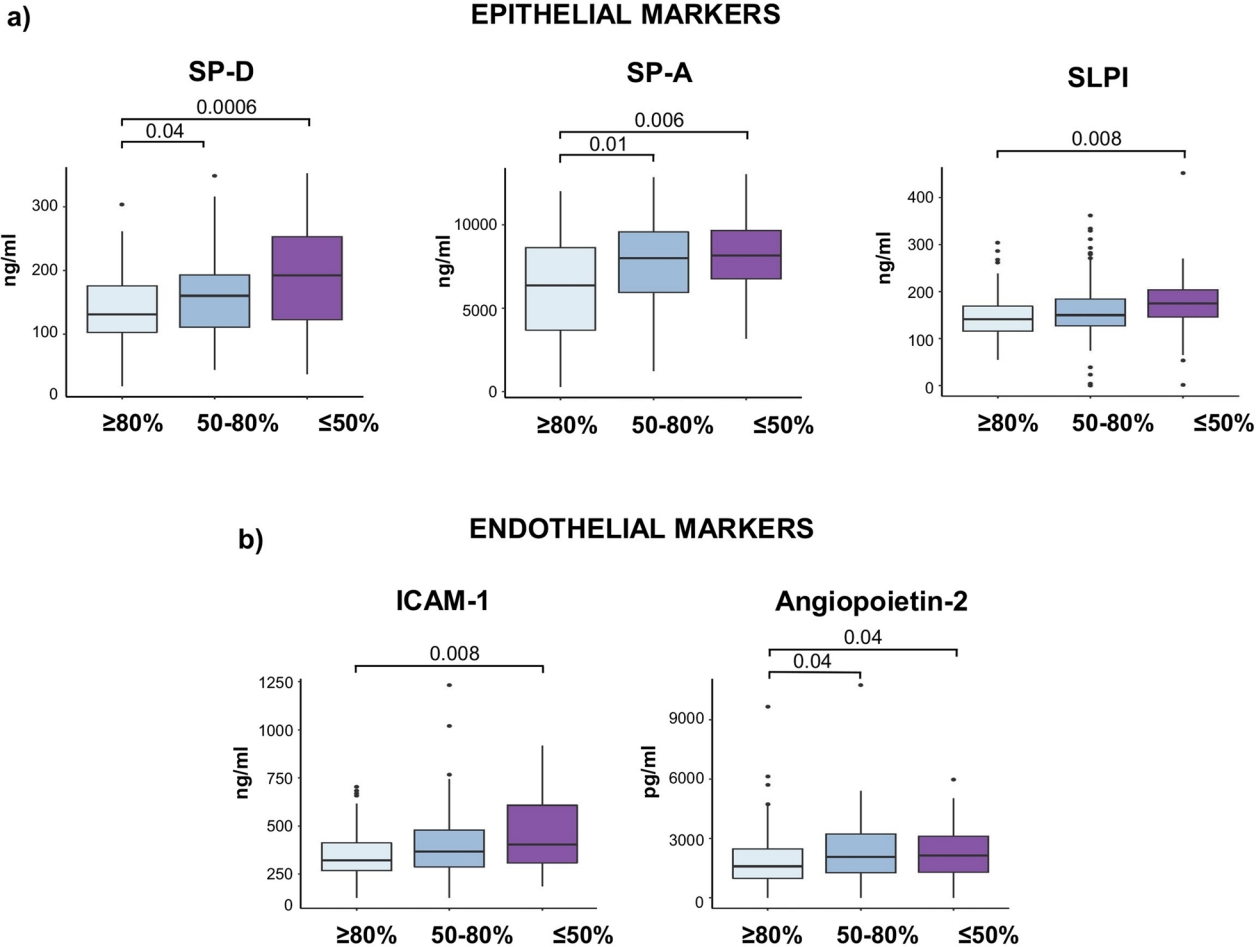
Association between epithelial and endothelial markers and the grades of DLCO alteration. **a** Levels of surfactant proteins (SP) A, SP-D and secretory leukocyte protease inhibitor (SLPI) as epithelial markers and **b** intercellular adhesion molecule 1 (sICAM-1) and Angiopoietin-2 as endothelial markers significantly increased in patients with the most abnormal DLCO (≤ 50%). Kruskal–Wallis tests are applied, and the adjusted *P*-values are obtained by Dunn’s test correction

Patients with reduced DLCO 6 months after discharge had significantly higher circulating levels of both epithelial (SP-A, SP-D, SLPI) and endothelial (sICAM-1 and Angiopoietin-2) markers, particularly in those with severely reduced DLCO (< 50% ref.) (Fig. 3). Significant associations also existed between DLCO values and these biomarkers (SP-A, rho = − 0.32, p < 0.001; SP-D, rho = − 0.25, p < 0.001; SLPI, rho = -0.15, p = 0.03; sICAM-1, rho = − 0.26, p < 0.001; and Angiopoietin-2, rho = − 0.15, p = 0.03) (see Additional file 1). Once patients that required mechanical ventilation were excluded from the analysis, SP-A (p = 0.004), SP-D (p = 0.002) and sICAM-1 (p = 0.025) still being significantly higher in patients with altered DLCO compared with patients with normal DLCO values. After this exclusion, significant associations still existing between DLCO values and SP-A (rho = − 0.32, p < 0.001), SP-D (rho = − 0.25, p = 0.001) and sICAM-1 (rho = − 0.24, p = 0.002). Also, when patients with comorbidities such as respiratory diseases, hypertension or diabetes were excluded, SP-A, SP-D and sICAM-1 remained significantly higher in this group of patients. When we analyzed the relationship of these markers with blood parameters, we observed weak but significant associations between sICAM-1 levels and LDH (rho = 0.3, p = 0.001) and D-dimer (rho = 0.23, p = 0.03) levels. No differences in CC16 [ng/ml, 24 (14.3–35.4) vs 22.5 (14.8–36.4), p = 0.7] and sE-selectin [ng/ml, 12.7 (6.5–25.6) vs 12.1 (6.99–25.5), p = 0.8] levels were found among patients with normal and reduced DLCO.

**Fig. 4:**
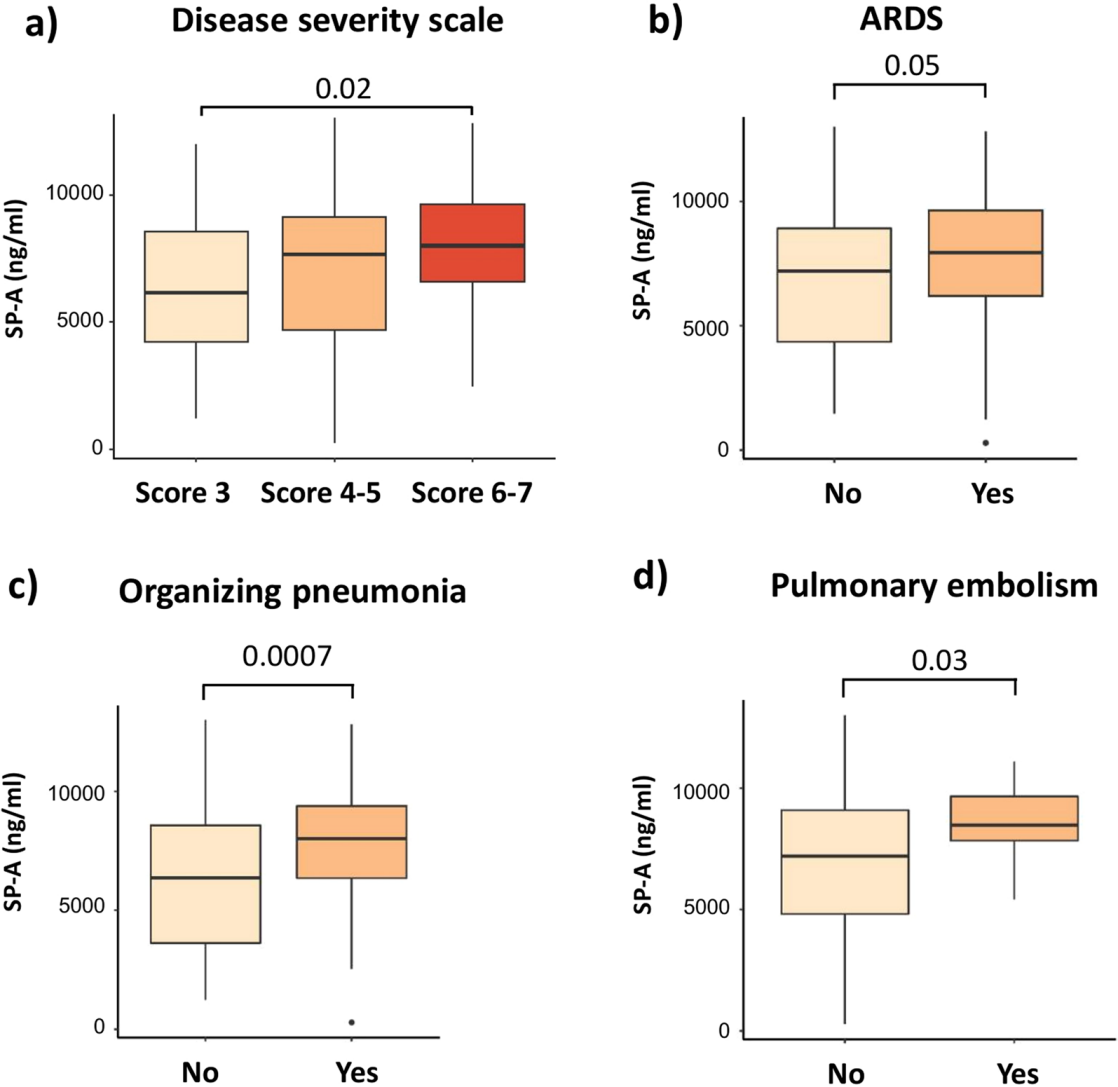
Association between epithelial markers and clinical severity during hospitalization. Surfactant protein (SP) A levels according to **a** the severity scale, **b** acute distress respiratory syndrome (ARDS), **c** the development of organizing pneumonia and **d** pulmonary embolism. Kruskal–Wallis test or Mann–Whitney test are applied, as appropriate. The adjusted *P*-values are obtained by Dunn’s test correction

Finally, we explored if the levels of these circulating markers during the follow-up were related to the acute COVID-19 event. Figure 4 shows that SP-A levels were higher in patients with more severe acute disease (WHO scores 6 and 7) or in those who had suffered ARDS, organizing pneumonia and pulmonary embolism during hospitalization. Additionally, patients who suffered ARDS had higher levels of SLPI (ng/ml, 160.6 (138.8–204) vs 143.6 (121.5–175.6), p = 0.008) and patients who develop organizing pneumonia had higher levels of SP-D (ng/ml, 160.7 (116.6–200.8) vs 147.3 (102–185), p = 0.048). Of note, endothelial biomarkers levels during follow-up were not related with events during hospitalization.

## Discussion

This study shows that, 6 months after discharge, reduced DLCO is common in COVID-19 survivors, that age older than 60 years and mechanical ventilation requirement are significant risk factors, and that these patients present elevated systemic levels of several circulating markers of lung epithelial and endothelial damage. Collectively, these observations suggest that epithelial and endothelial lung damage persist in severe COVID-19 patients for at least 6-months after hospital discharge. These results have a direct relationship with DLCO values, although whether these are permanent or reversible changes, and whether this is cause or consequence of COVID-19, require follow-up of these patients.

### Previous studies

Our clinical and functional findings here are in keeping with previous studies by our group and others evaluating DLCO after hospital discharge in COVID-19 survivors [3–8]. They are also in line with the reduction in DLCO observed in non-COVID-19-related ARDS survivors at 6- and 12-months after hospital discharge [31, 32]. Although recent studies have suggested cardiopulmonary recovery after COVID-19 [9], our findings indicate that a subgroup of patients (≥ 60 years requiring mechanical ventilation and/or additional organ support during admission because of acute COVID-19) still present reduced DLCO 6 months after discharge. The pathophysiological causes for persistent alterations in gas-blood exchange are not fully elucidated yet, but reduced DLCO may suggest interstitial or pulmonary vascular alterations caused by COVID-19 [3]. These structural lung changes may damage diffusion membrane, especially in those patients with severe disease. Their future follow-up will help to elucidate if these alterations are permanent or resolve with time.

### Interpretation of novel findings

It has been recently recommended that clinical observational studies on post-COVID-19 conditions investigate biomarkers after, at least, 6 months post-hospital discharge [33]. To our knowledge, this is the first study to do so. We found that the epithelial markers SP-A, SP-D and SLPI are increased in patients with abnormal DLCO 6 months after hospital discharge, reflecting a persistent epithelial injury. SP-A and SP-D are surfactant-related soluble pattern recognition receptors mainly produced by alveolar-epithelial type II cells (AEC-II) and involved in the lung innate immune response against viral and bacterial pathogens [34]. Some previous studies have reported elevated serum SP-D levels during the acute COVID-19 episode and suggested that this may be protective [35–37]. Although Club cells can both secrete CC16 and surfactant proteins, CC16 levels were not associated with reduced DLCO in COVID-19 patients. These results may suggest that AEC-II cells are one of the key cells involved in the pathophysiology of reduced DLCO in COVID-19 survivors. Interestingly, SP-A also participates in the protease and antiprotease activity through the regulation of SLPI [38], suggesting that the elevated SLPI levels observed in patients with reduced DLCO may be induced by the high SP-A concentrations.

Regarding the endothelial markers, although sICAM-1 and Angiopoietin-2 are not specific biomarkers for pulmonary vasculature, we found that their levels were increased in patients with abnormal DLCO 6-months after hospital discharge. Both markers are elevated during the acute phase of SARS-CoV-2 infection in relation to disease severity [17–19]. sE-selectin was found to be increased during the acute phase, especially in COVID-19-related ARDS [17], but we did not observe any significant relationship with reduced DLCO at 6-months after hospital discharge. This fact suggests that sE-selectin may have a role only during the initial steps of the pulmonary vasculature damage. Therefore, our findings highlight a persistent endothelial injury in patients with lung sequelae. However, there is a temporal evolution of the endothelial dysfunction markers during the progression of the disease that it should be considered. Viecelli Dalla Sega F. et al.showed that the time course for some endothelial dysfunction markers was different between COVID-19 survivors and non-survivors [39]. Further studies evaluating the levels of these markers during an extended follow-up are needed to know their stability over time.

We also observed, as other works, that patients with mild and moderate COVID-19 can also suffer long-term *sequelae* [33]. In fact, several of the biomarkers quantified here, including SP-D, SP-A and sICAM-1 still being higher after excluding by mechanical ventilation requirement. Also, these markers still being significantly different when patients with hypertension and/or diabetes are excluded, suggesting that our results are not biased by the comorbidities of the population. In contrast, we observed that SLPI and Angiopoietin-2 did not remain associated with reduced DLCO after mechanical ventilation exclusion, suggesting that they are markers dependent of ventilator-induced lung damage. However, the associations shown in our study may not be causative, and further studies are needed to address this question.

### Potential limitations

Our study has several limitations. First, DLCO measurements were not available before COVID-19, so we cannot discard that the patients studied here might have reduced DLCO before suffering COVID-19. However, this is unlikely because the proportion of patients with previously known respiratory diseases in our cohort was very small. Second, data about additional explorations at 6-months were not available for the analysis. Third, we did not include a control group in our study, so we do not know if the levels determined in the patients studied here are normal or not. Yet, it is unclear what type of control individuals should be studied here, healthy ones or survivors of other acute infective episodes. Finally, these biomarkers were not measured during the admission nor at hospital discharge but 6 months later. Future studies will have to validate them as potential prognostic markers of lung *sequelae* after discharge.

## Conclusions

This study shows that reduced DLCO is frequent in COVID-19 patients 6 months after hospital discharge, particularly in those older than 60 years who required mechanical ventilation during admission. We also showed that several circulating epithelial and endothelial pulmonary markers are increased in patients with reduced DLCO. All in all, our results indicate that the pulmonary injury induced by SARS-CoV-2 can leave pulmonary damage six months after hospital discharge. Future studies will have to investigate if this damage is transient or permanent.

## Supplementary Information

**Additional file 1: Fig. S1**. Additional correlations for DLCO values. Graphs representing the Spearman’s rank correlations between DLCO values and epithelial and endothelial biomarkers measured in COVID-19 survivors at 6-months after hospital discharge

## Data Availability

The datasets used and/or analysed during the current study are available from the corresponding author on reasonable request.

## Abbreviations

AEC-II: Alveolar type II epithelial cells
ARDS: Acute respiratory distress syndrome
CC16: Club cell secretory protein-16
CI 95%: 95% Confidence interval
COPD: Chronic obstructive pulmonary disease
CRP: Protein C reactive
DLCO: Carbon monoxide lung diffusion capacity
EDTA: Ethylenediaminetetraacetic acid
FEV_1_: Forced expiratory volume in 1st second
FVC: Forced vital capacity
sICAM-1: Soluble intercellular adhesion molecule 1
ICU: Intensive care unit
IQR: Interquartile range
RRT: Renal replacement therapy
ECMO: Extracorporeal membrane oxygenation
LDH: Lactate dehydrogenase
MV: Mechanical ventilation
NIV: Non-invasive mechanical ventilation
OR: Odds ratio
SD: Standard deviation
SLPI: Secretory leukocyte protease inhibitor
SP-A: Surfactant protein A
SP-D: Surfactant protein D
