# Supplementary figures and images for: Elevated plasma levels of epithelial and endothelial cell markers in COVID-19 survivors with reduced lung diffusing capacity six months after hospital discharge
Source: Respir Res. 2022 Feb 21;23:37. doi: 10.1186/s12931-022-01955-5 (PMC8860292; doi:10.1186/s12931-022-01955-5)

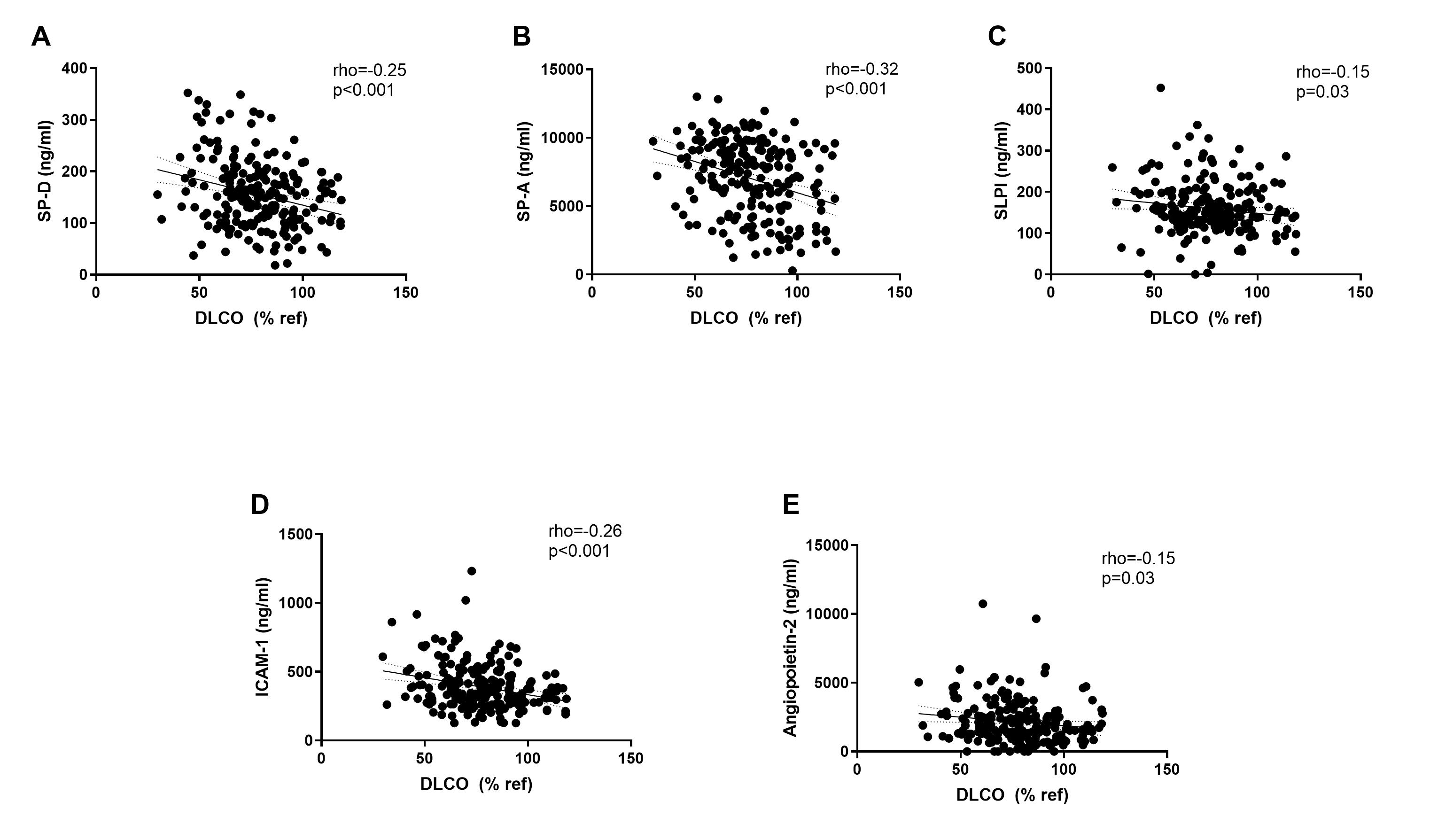

Supplement: Supplementary file 1 — Additional file 1: Fig. S1. Additional correlations for DLCO values. Graphs representing the Spearman’s rank correlations between DLCO values and epithelial and endothelial biomarkers measured in COVID-19 survivors at 6-months after hospital discharge [file 12931_2022_1955_MOESM1_ESM.jpg]
